# The impact of vitamin D supplementation on musculoskeletal health outcomes in children, adolescents, and young adults living with HIV: A systematic review

**DOI:** 10.1371/journal.pone.0207022

**Published:** 2018-11-15

**Authors:** Justin Penner, Rashida A. Ferrand, Ceri Richards, Kate A. Ward, James E. Burns, Celia L. Gregson

**Affiliations:** 1 University of Manitoba, Winnipeg, Canada; 2 Clinical Research Department, London School of Hygiene and Tropical Medicine, London, United Kingdom; 3 Department of Paediatrics, University of British Columbia, Vancouver, Canada; 4 Biomedical Research and Training Institute, Harare, Zimbabwe; 5 MRC Lifecourse Epidemiology, University of Southampton, Southampton, United Kingdom; 6 Queen Elizabeth University Hospital, Greater Glasgow & Clyde NHS Trust, Glasgow, United Kingdom; 7 Musculoskeletal Research Unit, Translational Health Sciences, Bristol Medical School, University of Bristol, Bristol, United Kingdom; Rush University, UNITED STATES

## Abstract

**Objective:**

HIV-positive children, adolescents, and young adults are at increased risk poor musculoskeletal outcomes. Increased incidence of vitamin D deficiency in youth living with HIV may further adversely affect musculoskeletal health. We investigated the impact of vitamin D supplementation on a range of musculoskeletal outcomes among individuals aged 0–25 years living with HIV.

**Methods:**

A systematic review was conducted using databases: PubMed/Medline, CINAHL, Web of Knowledge, and EMBASE. Interventional randomised control trials, quasi-experimental trials, and previous systematic reviews/meta-analyses were included. Outcomes included: BMD, BMC, fracture incidence, muscle strength, linear growth (height-for-age Z-score [HAZ]), and biochemical/endocrine biomarkers including bone turnover markers.

**Results:**

Of 497 records, 20 studies met inclusion criteria. Thirteen studies were conducted in North America, one in Asia, two in Europe, and four in Sub-Saharan Africa. High-dose vitamin D supplementation regimens (1,000–7,000 IU/day) were successful in achieving serum 25-hydroxyvitamin-D (25OHD) concentrations above study-defined thresholds. No improvements were observed in BMD, BMC, or in muscle power, force and strength; however, improvements in neuromuscular motor skills were demonstrated. HAZ was unaffected by low-dose (200–400 IU/day) supplementation. A single study found positive effects on HAZ with high-dose supplementation (7,000 *vs* 4,000IU/day).

**Conclusions:**

Measured bone outcomes were unaffected by high-dose vitamin D supplementation, even when target 25OHD measurements were achieved. This may be due to: insufficient sample size, follow-up, intermittent dosing, non-standardised definitions of vitamin D deficiency, or heterogeneity of enrolment criteria pertaining to baseline vitamin D concentration. High-dose vitamin D may improve HAZ and neuromuscular motor skills. Adequately powered trials are needed in settings where HIV burden is greatest.

PROSPERO Number: CRD42016042938.

## Introduction

The global scale-up of antiretroviral therapy (ART) has dramatically improved survival of those living with HIV and converted what was once a life-threatening infection into a chronic, treatable condition. HIV management now includes treatment of HIV infection, as well as associated chronic comorbidities, for example increased risk of low bone mineral density (BMD) [1–4]. Low BMD in youth living with HIV has been shown to far exceed those of HIV-negative controls [3,5]. Similarly, multiple observational studies, in both high- and low-middle-income countries (LMIC), have demonstrated vitamin D insufficiency in HIV-positive children, adolescents, and young adults [6–9], with a single study demonstrating increased rates compared to HIV-negative age-matched controls [10].

During childhood and adolescent growth, bones grow in length, width and mineral content until peak bone mass (PBM) is achieved [11]; PBM is a key determinant of future adult osteoporosis and lifetime fracture risk [12–14]. ‘Low bone mass’, defined as a dual energy X-ray absorptiometry (DXA) measured BMD Z-score ≤ -2 has been associated with low 25OHD and altered vitamin D metabolism in HIV-positive youths [15,16]. Furthermore, HIV infection increases bone turnover to reduce BMD even when vitamin D concentration is adequate [16]. HIV-associated alterations in vitamin D and bone metabolism are thought to arise from inflammatory and metabolic properties of the HIV infection itself [17–20] and/or side effects of ART [21–27] altering the molecular balance between bone formation and resorption. Intestinal absorption, nutritional intake and/or sun exposure may also be reduced [28–31]. HIV can cause delayed puberty [32,33] with associated reductions in bone mass [34–36] and restricted linear growth [37]. Inadequate dietary vitamin D is associated with growth failure (*i*.*e*. height-for-age Z-scores [HAZ] <-2) in HIV-positive children [38]. In sum, direct and indirect effects of HIV on musculoskeletal health are multifactorial and are potentially exacerbated by inadequate vitamin D.

Most current guidelines define vitamin D deficiency and insufficiency as a serum vitamin D value (25OHD) <30 nmol/L (<12 ng/ml) and between 30–50 nmol/L respectively (12–20 ng/ml); however, consensus is lacking and defined thresholds vary between countries and specialty advisory committees [39–41]. Some experts advocate higher values (e.g >75 nmol/L [>30 ng/ml]) in order to acieve maximal suppression of parathyroid hormone (PTH) and to optimise bone matrix formation in light of altered vitamin D metabolism with HIV [42].

Vitamin D supplementation has been shown to improve BMD in a range of paediatric chronic diseases such as epilepsy [43], kidney disease [44] and juvenile idiopathic arthritis [45], and to improve muscle function in HIV-negative adolescent girls [46]. Hence, it has been hypothesised that the beneficial effects of vitamin D supplementation can be reproduced in HIV infection.

Globally, there is an absence of evidence-based guidelines for vitamin D supplementation in youth living with HIV. We aimed to systematically review the current evidence examining the relationships between vitamin D supplementation and a range of musculoskeletal outcomes in children, adolescents, and young adults living with HIV, to guide future strategies to optimise musculoskeletal health.

## Methods

### Search strategy

**Fig 1:**
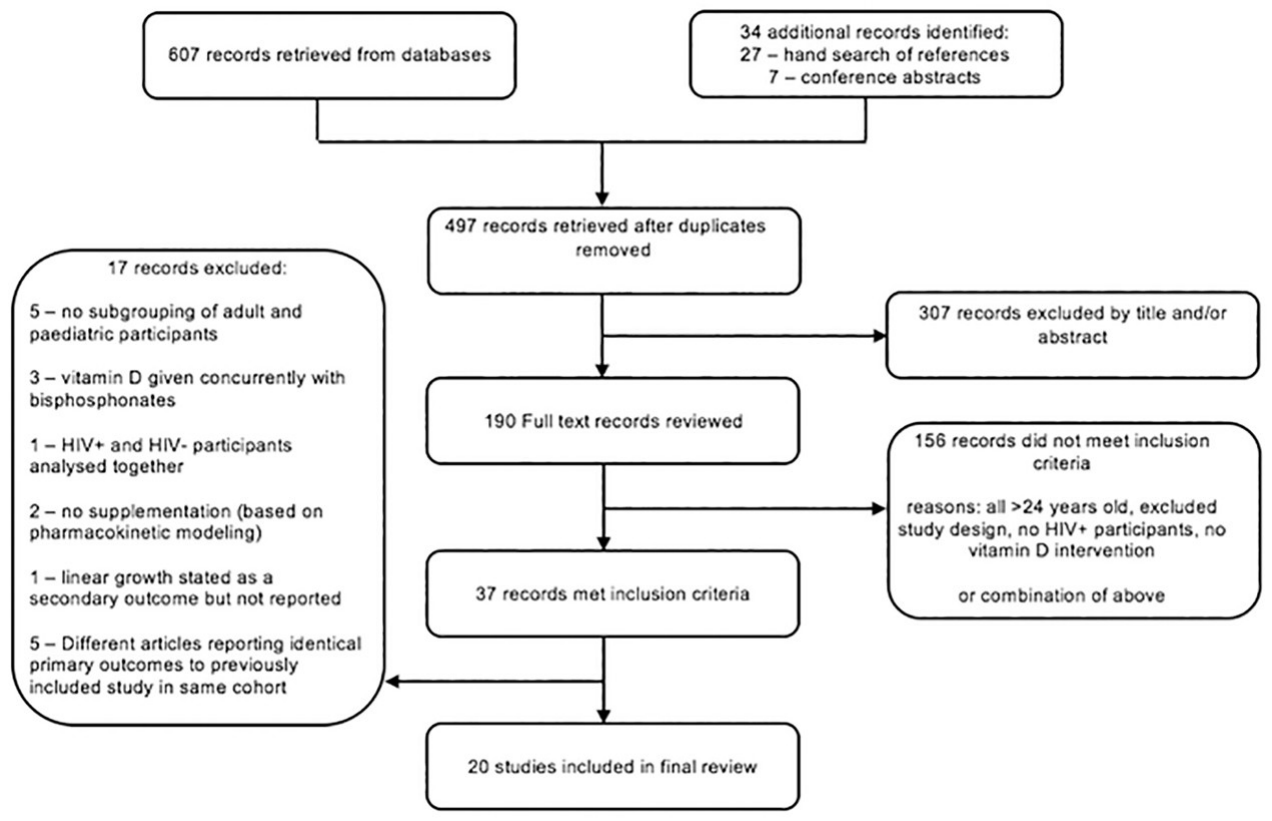
PRISMA flow diagram of search results.

Search strategy followed PRISMA guidance [47] (Fig 1). Articles were restricted to those published in English and French from the year 2000–2017, reflecting the period of ART availability. Articles were not restricted by geographic location. The following databases were searched: PubMed/Medline, EMBASE, CINAHL, and Web of Knowledge, (S1–S4 Tables). A hand search of the references cited for each retrieved article was performed. Furthermore, available conference abstracts, within the past six years, were reviewed from: (i) the Conference on Retroviruses and Opportunistic Infections, (ii) International AIDS Society, (iii) Infectious Diseases Society of America ID Week, and (iv) the American Society for Bone and Mineral Research.

### Study selection

Interventional trials of oral or parenteral vitamin D with or without calcium were assessed, including randomised control trials (RCT) and quasi-experimental studies (QET) (both controlled and uncontrolled). Controlled QET consisted of pre- and post-intervention studies with vitamin D and non-vitamin D arms, in addition to studies comparing high-dose and standard-dosing regimens (standard-dose defined: ≤800 International Units [IU]/day). Uncontrolled QETs were considered if they utilised either a one group pre- post-intervention design (vitamin D only) or an interrupted-time-series design [48]. Previous systematic reviews were included. To ensure enough time to achieve outcomes, a minimum of six months follow-up after enrolment was required for studies reporting radiological or clinical outcomes and three months for those reporting biochemical/endocrine outcomes. Studies that had at least 10 individuals aged 1 month to 25 years, regardless of the mode of HIV acquisition and HIV treatment status (ART naïve or experienced) were considered. Studies assessing bisphosphonates in conjunction with vitamin D supplementation were excluded.

Eligibility for inclusion was determined independently by two reviewers (JP & CR) using an assessment toolkit with a pre-defined inclusion checklist [49,50].

### Data extraction and quality assessment

Data were extracted independently using the Cochrane Public Health Group Data Extraction and Assessment Form [50], with risk of bias (ROB) assessed using the Cochrane Handbook for systematic reviews and reported independently as high, low, or unclear by both reviewers (JP & CR) [49]. Data on study latitude/geographic location, participant age, gender, seasonality, adherence, dietary vitamin D intake (IU/day), skin colour/sun exposure and method of 25OHD and 1,25OHD quantification were recorded.

### Biochemical/endocrine outcomes

Data were extracted for the biologically active variant and the stored form of vitamin D, hydroxylated (1,25OHD) and non-hydroxylated (25OHD) vitamin D respectively, as well as: endocrine/biochemical markers (calcium, phosphate, bone alkaline phosphatase [BAP], PTH, and fibroblast growth factor [FGF]), serum and urine biomarkers of bone turnover (osteocalcin [OC], procollagen type-1 N-terminal propeptide [P1NP], collagen type-1 cross-linked C-telopeptide [CTX], and N-terminal telopeptide [NTX]), and markers of systemic inflammation (CRP, D-dimer, interleukins, tumour necrosis factors, and interferons).

Doses of vitamin D supplementation were standardised into daily doses (IU/day). Definitions of vitamin D deficiency, insufficiency, and sufficiency were extracted for between-study comparison (S5 Table).

### Musculoskeletal outcomes (bone, muscle and linear growth)

BMC and BMD measured using the following radiographic techniques were acceptable: DXA, computed tomography (including pQCT and high resolution pQCT), quantitative ultrasound (measured by either speed of sound, broadband ultrasound attenuation, or stiffness index), and/or digital X-ray radiogrammetry. For those not reporting BMD Z-scores, BMD measurements were compared against standardised, age-matched paediatric Z-scores as outlined by the International Society of Clinical Densitometry (ISCD) [51].

Studies assessing muscle function by measuring muscle strength, power, and/or force were considered relevant, as were studies assessing muscle size and mass. Linear growth was reported as HAZ, where SDs and standard error can be computed from *Z*-score values. HAZ summary statistics by age/sex represent a comparison to WHO reference standards with an expected mean HAZ of zero and SD of one. Z-scores were compared to the WHO Global Database on Child Growth and Malnutrition standard classifications, if not reported by the study authors [52]. Clinical outcomes of incident fractures and prevalent rickets and osteomalacia were extracted.

### Adverse events

All adverse effects of vitamin D supplementation were recorded. Hypercalcemia, hypercalciuria, renal calculi, and gastrointestinal upset were specifically screened for as these are recognised complications of cholecalciferol supplementation in healthy populations [53].

## Results

### Study characteristics

A total of 607 articles were retrieved from database searches, a further 27 from hand searches of references, and 7 conference abstracts. After removal of duplicates, 497 articles were screened by title and abstract. The resulting 190 articles were then reviewed in full, of which 37 studies met inclusion criteria (Fig 1). The final 20 included studies were published between 2009–2017. Thirteen studies were conducted in North America (USA, Canada), two in Europe (France, Italy), four in Sub-Saharan Africa (Botswana, South Africa, Uganda), and one published conference abstract from Thailand. The follow-up time of the studies ranged from three months to two years. All studies used oral Vitamin D_3_ (cholecalciferol) except the ongoing Sudjaritruk *et al*. study which used D_2_ (ergocalciferol) for supplementation [54]. Four studies supplemented calcium in conjunction with vitamin D [55,56,57,58] whereas three studies utilised a multiple-micronutrient supplement which did not contain calcium [59,60,61]. There was considerable dosing heterogeneity in regimens, ranging from 200–7,000 IU/day. Lower dose regimens (200–400 IU/day) were used primarily in studies measuring HAZ, whereas higher doses (1,000–7,000 IU/day) were used in those measuring biochemical/endocrine and bone/muscle outcomes.

Studies are listed in Tables 1–4. Brown *et al*. [62] and Rovner *et al*. [63] published a secondary analyses of muscle and bone outcomes generated by Stallings *et al*. [64]. Arpadi *et al*. (2012) published a follow-up study of the same population described by Arpadi *et al*. (2009), but outcomes differed [55,56]. Havens *et al*. (2012b & 2014) published two secondary analyses of data reported initially by Havens *et al*. (2012a) [65–67] and a third study assessing BMD in conjunction with longitudinal biochemical data [68].

**Table 1:** Characteristics of studies assessing serum biomarkers/endocrine factors in response to cholecalciferol supplementation.

Study author, year, country	Study design	Population (n) and gender^6^	Age^1^ range and mean (SD)	Mode of HIV acquisition	Intervention^2^ (mean daily VD dose)^3^ (n)	Control (n)	Baseline 25OHD (nmol/L)^4^ and exclusions	Summary of main findings (25OHD nmol/L)^4^^,^^5^
**Arpadi et al., 2009,** **USA**[55]	Double-blind RCT	56 M: 39.3%	6–16 years	100% perinatal	100,000 IU bimonthly^7^ +1g Ca/day (= 1,667 IU/day) (n = 29)	Double placebo (n = 27)	Baseline 25OHD in VD+ = 62.2 (22.7)	Higher mean serum 25OHD in VD+ (p<0.0001)Trough serum 25OHD higher in VD+ Higher monthly 25OHD in VD+ group (p<0.001)
	Stratified by gender and age		VD+ 10.2(2.9) VD- 10.6 (2.4)				25OHD <30 nmol/L excluded (n = 5)	
							Participants on TDF excluded	
**Dougherty et al., 2014,** **USA** [72]	Double-blind uncontrolled QET (pre- post-intervention experimental trial)	44 M: 18.7%	8.3–24.9 years 18.7(4.7)	43% perinatal 57% horizontal	4,000 IU/day (n = 22) 7,000 IU/day (n = 22)	None	Baseline 25OHD in 4,000 IU/day = 18.6 (11–81.1)^9^	A priori criteria for efficacy: >80% achieving 25OHD ≥79.9 nmol/L Sufficient 25OHD achieved in 76% of 4,000 IU/day vs. 86% in 7,000 IU/day group Increased 25OHD/1,25OHD for both groups (both, p<0.05) Decrease PTH only in 4,000 IU/day (p<0.005)
			4,000 IU group 18.4 (4.5) 7,000IU group 19.1 (5)				Baseline 25OHD in 7,000 IU/day = 56.2 (28.2–83.9)^9^	
							No baseline 25OHD exclusion criteria	
**Eckard et al., 2017,** **USA** [77]	Double-blind RCT	102 M: 64%	8–25 years 20.3 (16.6–22.8)^8^	53% perinatal	60,000 IU monthly 120,000 IU monthly (= 2,000 IU/day 4,000 IU/day) (n = 36)^9^	18,000 IU monthly (= 600 IU/day) (n = 66)	Baseline 25OHD for all participants = 42.4 (32.4–54.9)^8^	Only 120,000IU arm decreased P1NP (p = 0.001) and CTX (p = 0.0005), No change in OC Increase in 25OHD in both VD+ (p<0.001) and VD- (p<0.001) with significant intergroup difference (p = 0.001) No change in PTH in any group
	Initially stratified by EFV use						Excluded if baseline 25OHD >75	
**Foissac et al., 2014,** **France** [69]	Open-label QET (pre- post-pharmacokinetic intervention trial)	91 M: 51.6%	3–24 years	Not Reported	100,000 IU every 3 months (= 1,096 IU/day) (91)	None	Baseline 25OHD for all participants– 30 (22.5–42.4)^8^	Lower 25OHD after 12 months of VD when baseline 25OHD <25 vs. 25OHD 50–75: 62.4(37.4,67.4)^7^ vs. 102.3(49.9,167.2)^7^ 6 months of VD needed for 50% of participants with baseline 25OH<25 to reach 25OHD >74.9 vs 3.4 months when baseline 25OHD 25–74.9 Optimal dosing regimen when baseline 25OHD <25 = 100,000IU/day x 2 doses (day 1&15) then 100,000 IU every 3 months starting at day 30 By modeling, most effective regimen = 50,000 IU/month or 100,000IU bimonthly^5^
			reported by sex: boys: 14(10–17)^8^ girls: 15(11–17)^8^				No baseline 25OHD exclusion criteria	
**Giacomet et al., 2013,** **Italy** [70]	Double-blind RCT	52 NR	8–26 years	100% perinatal	100,000 IU every 3 months (1,096IU/day) (n = 26)	placebo oil (n = 26)	Baseline 25OHD for VD+ = 37.4 (30–47.4)^8^	VD insufficiency 60% in VD- vs. 20% in VD+ at 12 months (p = 0.007) Higher mean change in 1,25OHD at 12 months in VD+ (p<0.001)
			VD+ 20 (14–23) ^7^ VD- 18 (15–23) ^8^				25OHD >74.9 and PTH above normal limits excluded Excluded black ethnicity	
**Havens et al. 2012a,** **USA & Puerto Rico** [66]	Double-blind RCT	203 M: 62.6%	18–24.9 years 20.9(2)	“predominantly (perinatal)” (% not defined)	50,000 IU/month (= 1,667 IU/day) TDF/VD+ (n = 59) “noTDF”/VD+ (n = 43)	Placebo capsule TDF or placebo (n = 59) “no TDF” or placebo (n = 42)	Baseline 25OHD for all participants = 52.9 (30.7) No baseline 25OHD exclusion criteria	At 12 weeks decrease VD insufficiency/deficiency 52% to 5% (p<0.001) in VD+ Increased 25OHD & 1,25OHD in VD+ (both, p<0.001) with largest response in those with lowest baseline 25OHD (p = 0.019) No change in 25OHD/1,25OHD in VD- PTH (p<0.031), BAP (p = 0.038) decreased only in TDF/VD+ group No significant change in CTX or phosphate in any group Increase in FGF23 (p = 0.002) only in TDF/VD+ group
**Havens et al. 2012b** [67]	Stratified by TDF vs. “noTDF” then randomised		VD+ 20.9 (2.1) VD- 20.9 (1.9)					
**Havens et al. 2014** [65]								
**Havens et al., 2017** **USA & Puerto Rico** [57]	Double-blind RCT	214 M: 84%	16–24 years 22 (21–23)^7^	Not defined Median time since HIV diagnosis = 2 years	50,000 IU monthly + daily multivitamin of 400IU/day + 162mg Ca (2,067IU/day) (n = 109)	Placebo + multivitamin of 400 IU/day + 162 mg Ca (n = 105)	Baseline 25OHD for all participants = 40.9 (28.5–59.7)^8^	Greater increase in 25OHD (p<0.001) and greater change from baseline 25OHD (p = 0.001) and 1,25OHD (p = 0.014) in VD+ Sustained decrease in PTH in VD+ (p = 0.016) Sustained increase in FGF23 in VD+ and VD- (both 0.001>p-value <0.01) but no intergroup differences (p = 0.71) Sustained decrease in VD+ and VD- for BAP (both p<0.001), OC (both p<0.001), CTX (VD+ 0.001>p-value <0.01; VD- p<0.001) but no intergroup differences No change in Ca or phosphate
	Participants all on TDF containing ART Stratified by sex, age, and race						No baseline 25OHD exclusion criteria	
**Kakalia et al., 2011,** **Canada** [73]	Open-label RCT	53 M: 45.3%	3–18 years	91% perinatal 9% horizontal	11,200 IU/week (= 1,600 IU/day) (n = 18) or 5,600 IU/week (= 800 IU/day) (n = 18)	None (n = 17)	Baseline 25OHD in 800 IU/day group = 49.9 (22.5) Baseline 25OHD in 1,6000 IU/day group = 42.7 (18.1)	Increased 25OHD for 5,600 IU/week (p = 0.0002) and 11,200 IU/week (p<0.001), not in VD- (p = 0.27) Higher mean increase 25OHD for 11,200 IU/week vs. 5,600 IU/week (p = 0.02) Only 67% of those supplemented achieved sufficient 25OHD No significant changes in 1,25OHD (p = 0.9), PTH (p = 0.9), calcium, or phosphate (no p-values)
			800IU group 10.6 (4.4) 1600IU group 10.3 (3.2) VD- 10.7(4.4)				25OHD <25 nmol/L excluded	
**Poowuttikul et al., 2014,** **USA** [76]	Open-label uncontrolled QET (single-arm pre-post-intervention design)	160 M: 76.3%	2–26 years	Not reported	1,000 IU/day (n = 152)	None	23.1% of participants with baseline 25OHD 50–87.4 71.9% of participants had baseline 25OHD <49.9 Only participants with 25OHD <87.5 were supplemented	39.5% of VD+ showed improvement in 25OHD Of those with improved 25OHD 16.7% achieved 25OHD >87.4 nmol/l
			8.1% ≤10 years (n = 13) 45% 11–20 years (n = 72) 26.9% 21–26 years (n = 75)					
**Stallings et al., 2015,** **USA** [64]	Double-blind RCT	58 M: 38.9%	5–24.9 years 20.7(3.7)	36% perinatal 64% horizontal	7000 IU/day (n = 30)	Placebo pill/liquid drops (n = 28)	Baseline 25OHD for all participants = 43.9 (21.7)	Significant difference between VD+ and VD- participants exceeding 25OHD >79.9 (p<0.01), >49.9 (p<0.001), and >27.5 (p<0.02) No change in PTH
	Stratified by mode of HIV acquisition then randomised		VD+ 21.3 (3.3) VD- 20.0 (4.1)				Participants with 3 consecutive 25OHD <27.5 nmol/L withdrawn	
**Steenhoff et al., 2015,** **Botswana** [71]	Double-blind uncontrolled QET (pre- post-intervention design)	60 M: 50%	5–50.9 years^10^ 19.5(11.8)	68% perinatal 32% horizontal	7,000 IU/day (n = 30) 4,000 IU/day (n = 30)	None	Baseline 25OHD in 7,000 IU/day group = 86.1 (23.7) Baseline 25OHD in 4,000 IU/day group = 91.1(23.2)	Intragroup differences in 25OHD (p<0.001, both groups), and PTH (p<0.01, p<0.05 4,000 IU/day and 7,000 IU/day respectively) No intergroup difference in 25OHD or PTH Participants 5–13 years had greatest rise in 25OHD (p<0.001)
	4,000IU group 19.5 (11.8) 7,000 group 19.5 (12)		Stratified into 5 age groups then randomised					
**Preliminary Interim Data (from published abstract)**								
**Sudjaritruk et al. 2017** **Thailand** [54]	Open-label randomized trial	166 M: 48%	10–20 years 16.0 (14.4–17.7)	100% perinatal Median duration of ART 10 years	“high-dose”: ergocalciferol/Ca (3200IU/1.2 g day)	“normal-dose”: ergocalciferol/Ca (400IU/1.2 g day)	Baseline 25OHD for all participants = 25.3 (20.7–33.2)	Intragroup change from baseline for 25OHD, ALP, PTH, CTX, P1NP in VD+ and VD- groups (p<0.05) No intergroup changes in 25OHD, CTX, P1NP, or BAP intergroup difference in PTH (p = 0.007)
							No baseline 25OHD exclusion criteria	

^1^. Mean age based on group allocation and/or overall age when reported.

^2^. All oral cholecalciferol.

^3^. Calculated based on 30 days/month.

^4^. Means (standard deviation) unless otherwise specified.

^5^. ng/ml transformed to nmol/L

^6^. Gender reported as percentage male

^7^. Bimonthly defined: once every 2 months.

^8^. Median (interquartile range).

^9^. 60,000 and 120,000 IU/month groups considered together except in bone turnover marker analysis

^10^. Age adjusted linear model for paediatric patients

1,25OHD = Serum 1,25-dihydroxyvitamin D_3_ level; 25OHD = Serum 25-hydroxyvitamin D_3_ concentration; BAP = Bone Alkaline Phosphatase; Ca = Calcium; CTX = Collagen Type-1 Cross-linked C-telopeptide; FGF23 = Fibroblast Growth Factor-23; NR = Not Reported; OC = Osteocalcin; P1NP = Procollagen Type-1 N-terminal Propeptide; PTH = Parathyroid Hormone; QET = Quasi-Experimental Trial; RCT = Randomized Control Trial; TDF = Tenofovir; VD+ = Vitamin D Intervention Arm; VD- = Control Arm

**Table 2:** Characteristics of included studies assessing bone outcomes in response to cholecalciferol supplementation.

Study author, year, country	Study design	Population (n) and gender^6^	Age^1^ range and mean (SD)	Mode of HIV acquisition	Intervention^2^ (mean daily VD dose)^3^ (n)	Control (n)	Baseline vitamin D (nmol/L)^4^ levels and exclusions	Summary of main findings (25OHD nmol/L)^4^^,^^5^
**Arpadi et al., 2012,** **USA** [56]	Double-Blind RCT	59 M: 44.1%	6–16 years	100% perinatal	100,000 IU bimonthly^7^ +1g Ca/day (= 1,667 IU/day) (n = 30)	Double placebo (n = 29)	Baseline 25OHD in VD+ = 62.2 (22.7)	All intragroup bone mass indices increased over time No intergroup difference in TBBMC (p = 0.5), TBBMD (p = 0.5), LSBMC (p = 0.6), LSBMD (p = 0.5) at any time point
	Stratified by gender and age		VD+10.2(2.8) VD- 11.0(2.3)				25OHD <30 nmol/L excluded (n = 5) Participants on TDF excluded	
**Eckard et al., 2017** **USA** [77]	Double-Blind RCT	102 M: 64%	8–25 years 20.3 (16.6–22.8)^8^	53% perinatal	60,000 IU 120,000 IU monthly (= 2,000 IUday 4,000 IU/day) (n = 36)^8^	18,000 IU monthly (= 600 IU/day) (n = 66)	Baseline 25OHD for all participants = 42.4 (32.4–54.9)^8^	Intragroup % change LSBMD (p = <0.001) % change HBMD (p = 0.03), and spine Z-scores (p = 0.005) in VD+ but only an Intragroup change in % HBMD (p = 0.002) in VD- No intergroup differences in % change SBMD (p = 0.3), % change HBMD (0.37), spine (p = 0.15) or hip Z-score (p = 0.7) Combined (VD+ and VD-) intragroup difference in % change SBMD (p<0.001) and spine Z-score
	Initially stratified by EFV use						Excluded if baseline 25OHD >75	
**Havens et al., 2017** **USA & Puerto Rico** [57]	Double-Blind RCT	214 M: 84%	16–24 years 22 (21–23)^8^	Not defined Median time since HIV diagnosis = 2 years	50,000 IU monthly + daily multivitamin of 400 IU/day +162mg Ca (2,067 IU/day) (n = 109)	Placebo + multivitamin of 400 IU/day + 162 mg Ca (n = 105)	Baseline 25OHD for all participants = 40.9 (28.5–59.7)^8^	No intergroup differences in LSBMD No intragroup difference in VD+ or VD- for lumbar LSBMD although trend towards an increase in the VD+ group (+1.17% [-0.75% to +2.74] p<0.001) No change in HBMD or TBBMD
	Participants all on TDF containing ART Participants stratified by sex, age, and race						No baseline 25OHD exclusion criteria	
**Rovner et al., 2017** **USA** [63]	Double-Blind RCT	58 M: 69%	5–24.9 years 20.9 (3.6)	35% perinatal	7,000 IU/day (n = 30)	Placebo (n = 28)	Baseline 25OHD for all participants = 45.4 (21.2)	No intra- or intergroup differences in TBBMD, LSBMD, TBBMC, or pQCT tibia
	Participants stratified by age and mode of HIV acquisition						No baseline 25OHD exclusion criteria	
**Preliminary Interim Data (from published abstract)**								
**Sudjaritruk et al. 2017** **Thailand** [54]	Open-label randomized trial	166 M: 48%	10–20 years 16.0 (14.4–17.7)	100% perinatal Median duration of ART 10 years	“high-dose”: ergocalciferol/Ca (3200IU/1.2 g day)	“normal-dose”: ergocalciferol/Ca (400IU/1.2 g day)	Baseline 25OHD for all participants = 25.3 (20.7–33.2)	Significant increases over 48 weeks in LSBMD Z-score in both ‘high-dose’ and ‘normal dose’ groups with low-BMD, but not normal baseline BMD. No between group differences in change in LSBMD
	Analysis stratified by baseline Low-BMD lumbar spine Z-score						No baseline 25OHD exclusion criteria	

^1^. Mean/Median age based on group allocation and/or overall age when reported as such.

^2^. All oral cholecalciferol unless otherwise stated

^3^. Calculated based on 30 days/month.

^4^. Means (standard deviation) unless otherwise specified.

^5^. ng/ml transformed to nmol/L

^6^. Gender reported as percentage male

^7^. Bimonthly defined: once every 2 months.

^8^. Median (IQR) 8. 60,000 and 120,000 IU/month groups considered together in statistical analysis

25OHD = Serum 25-hydroxyvitamin D_3_ concentration; Ca = Calcium; EFV = Efavirenz; HBMD = Total Hip Bone Mineral Density;; LSBMC = Lumbar Spine Bone Mineral Content; LSBMD = Lumbar Spine Bone Mineral Density; pQCT = peripheral quantitative computed tomography; RCT = Randomised Control Trial; SBMC = Spinal Bone Mineral Content; SBMD = Spinal Bone Mineral Density; TBBMC = Total Body Bone Mineral Content TBBMD = Total Body Bone Mineral Density; TDF = Tenofovir; VD+ = Vitamin D Intervention Arm; VD- = Control Arm

**Table 3:** Characteristics of studies assessing muscle outcomes in response to cholecalciferol supplementation.

Study author, year, country	Study design	Population (n) and gender^6^	Age^1^ range and mean (SD)	Mode of HIV acquisition	Intervention^2^ (mean daily VD dose)^3^ (n)	Control (n)	Baseline vitamin D (nmol/L)^4^ levels and exclusions	Summary of main findings (25OHD nmol/L)^4^^,^^5^
**Brown et al., 2015,** **USA** [62]	Double-Blind RCT	56 M: 67.9%	5–24.9 years 20.7(3.8)	34% perinatal 66% horizontal	7,000 IU/day (n = 29)	Placebo pill/liquid (n = 27)	Baseline 25OHD for all participants = 43.7 (21.1)	No difference in jump power [Watts] (p = 0.4), peak jump energy [peak jump height in cm/seconds to complete the jump] (p = 0.14), muscular forces (p = >0.4), muscular strength (p = 0.9) VD+ vs. VD- VD+ improved neuromuscular motor skils vs. VD- (p = 0.04)
			VD+ 20.0 (4.1) VD- 21.4(3.3)				2 participants from parent study excluded (Cerebral Palsy)	
**Rovner et al., 2017** **USA** [63]	Double-Blind RCT	58 M: 69%	5–24.9 years 20.9 (3.6)	35% perinatal	7,000 IU/day (n = 30)	Placebo (n = 28)	Baseline 25OHD for all participants = 45.4 (21.2)	No intra- or intergroup differences in muscle cross sectional area
	Participants stratified by age and mode of HIV acquisition						No baseline 25OHD exclusion criteria	

^1^. Mean age based on group allocation and/or overall age when reported.

^2^. All oral cholecalciferol.

^3^. Calculated based on 30 days/month.

^4^. Means (standard deviation) unless otherwise specified.

^5^. ng/ml transformed to nmol/L

^6^. Gender reported as percentage male.

RCT = Randomised Control Trial; VD+ = Vitamin D Intervention Arm; VD- = Control Arm.

**Table 4:** Characteristics of studies assessing linear growth in response to cholecalciferol supplementation.

Study author, year, country	Study design	Population (n) and gender^6^	Age^1^ range and mean (SD)	Mode of HIV acquisition	Intervention^2^ (mean daily VD dose)^3^ (n)	Control (n)	Baseline vitamin D (nmol/L)^4^ levels and exclusions	Summary of main findings (25OHD nmol/L)^4^^,^^5^
**Chhagan et al., 2010,** **South Africa** [61]	Double-Blind RCT	317 (HIV-positive n = 25) M: 60.9%	6–24 months NR	100% perinatal	MMS^7^ containing 5μg vitamin D (200 IU/day) (n = 12)	Vitamin A (n = 9) Vitamin A and Zinc (n = 11)	No baseline 25OHD data	No participants on ART at baseline (regional unavailability) Worse growth patterns in MMS arm No statistical analysis performed on HIV+ group for stunting due to small sample size, thus, only trend demonstrated
	Stratified by HIV-status and maternal exposure then randomised						No baseline 25OHD exclusion criteria	
**Mda et al., 2010,** **South Africa** [59]	Double-Blind RCT	99 NR	6–24 months	100% perinatal	MMS^7^ containing 5μg (= 200 IU/day) (n = 50)	Placebo powder dissolved in water (n = 49)	No baseline 25OHD data	No increase in HAZ at 6 months from baseline in VD+ group (no p-value) nor difference between VD+ and VD- (no p-value
			VD+ 15.1(5.4) VD- 13.6(5.7)				No baseline 25OHD exclusion criteria Participants on ART excluded	
**Ndeezi et al., 2010,** **Uganda** [60]	Double-Blind RCT	847 M: 50.3%	12–59 months	100% perinatal	Enhanced MMS^7^ containing 400 IU/day vitamin D (contains 14 micronutrients at 2 times RDA) (n = 426)	Standard MMS^7^ containing vitamin D 200 IU/day (6 micronutrients at RDA) (n = 421)	No baseline 25OHD data	No difference in HAZ (p = 0.08)
	Stratified by ART vs no ART then randomised Treatment group given enhanced MMS for 6 months then standard MMS for remaining 6 months of study		ART+ VD+ (n = 43) (8.6%)<36months ART+ VD- (n = 42) 38.1%<36months No ART+ VD+ (n = 383) 57.2%<36months No ART+ VD- (n = 379) 59.9%<36months				No baseline 25OHD exclusion criteria	
**Steenhoff et al., 2015,** **Botswana** [71]	Double-blind uncontrolled QET (pre- post-intervention design)	60 M: 50%	5–50.9 years^8^ 19.5 (11.8)	68% perinatal 32% horizontal	7,000 IU/day (n = 30) 4,000 IU/day (n = 30)	None	Baseline 25OHD in 7,000 IU/day group = 86.1 (23.7) Baseline 25OHD in 4,000 IU/day group = 91.1(23.2)	28% stunted at baseline HAZ different at 12 weeks from baseline only in 7,000 IU/day group (p<0.01)
	4,000 IU group 19.5 (11.8) 7,000 IU group 19.5 (12)		Stratified into 5 age groups then randomised					

^1^. Mean age based on group allocation and/or overall age when reported.

^2^. All oral cholecalciferol.

^3^. Calculated based on 30 days/month.

^4^. Means (standard deviation) unless otherwise specified.

^5^. ng/ml transformed to nmol/L

^6^. Gender reported as percentage male

^7^. Multivitamin did not contain calcium

^8^. HAZ subcategorized by age (<20 years old; n = 40)

25OHD = Serum 25-hydroxyvitamin D_3_ concentration; ART = Antiretroviral Therapy; HAZ = Height-for-Age Z-score; MMS = Multiple-micronutrient Supplement; NR = Not Reported; QET = Quasi-Experimental Trial; RCT = Randomized Control Trial; RDA = Recommended Daily Allowance; VD+ = Vitamin D Intervention Arm; VD- = Control Arm

### Study participants

Overall, the 20 trials included individuals aged six months to 25 years old. With the exception of one Hepatitis C co-infected individual in the Foissac *et al*. [69] study, participants had no concomitant acute and/or chronic disease, apart from HIV. There were no patients noted to have concurrent TB. Mean CD4 count, when reported, varied between 587 [66] and 1041 [60], and average CD4% from 27.8% [55] to 35.5% [70]. The majority of participants did not have an AIDS-defining illness, except in the Botswanan trial published by Steenhoff *et al*., where 57% had CDC category C disease [71] and in the ongoing Thai trial where 50.3% had WHO stage 3 or 4 disease [54]. The percentage of participants on ART was variable, ranging from 10 to 100%. In the study by Mda *et al*. those on ART were excluded altogether [59]. Low dietary vitamin D intake among participants as compared to the Institute of Medicine (IOM) recommended daily allowance (RDA) was ubiquitous across studies. Similarly, dietary calcium intake was well below the RDA in the four studies to report this [56,57,64,72].

### Study outcomes

The outcomes assessed can be broadly classified into four categories, with several studies reporting primary outcomes in more than one of the following categories: 14 studies assessed biochemical/endocrine parameters, most notably serum 25OHD concentration (Table 1), five studies reported bone outcomes (Table 2), two studies described muscle function and structure findings (Table 3), and four studies analysed HAZ (Table 4).

All studies, except Mda *et al*. [59], Chhagan *et al*. [61], and the abstract by Sudjaritruk *et al*. [54] reported measures of potential harm and/or side-effects of the intervention although none were powered for safety. No significant adverse events directly related to the intervention were observed apart from two cases of renal calculi, although one was remote from the intervention and the other part of the placebo group receiving only 400 IU/day of vitamin D. Minor adverse events, when reported, consisted of transient hypercalcemia and hypercalciuria.

#### Biochemical/endocrine outcomes

The majority of studies aimed to raise concentrations of 25OHD >75 nmol/L with the goal of maximal PTH suppression (S5 Table). Kakalia *et al*. demonstrated persistent hyperparathyroidism with serum 25OHD values between 50–75 nmol/L [73]. To achieve a measurement >75 nmol/L, higher supplementation doses (>1,000 IU) than currently recommended were required; though it should be noted that other guidelines such as the Special Advisory Committee on Nutrition (SACN) [40], IOM [74], and European Food Safety Agency [75] guidlines are to achieve population mean levels above the threshold not intended for clinical purposes. Higher mean and trough serum 25OHD values were almost always achieved after supplementation [54,55,57,64,66,70–73,76,77]. Higher mean monthly 25OHD values were seen with higher doses of cholecalciferol [55]. Lower baseline values of 25OHD negatively impacted final serum 25OHD concentrations, and extended the time needed to reach 25OHD >50 nmol/L and 75 nmol/L [66,69]. Without supplementation, dietary intake of cholecalciferol, noted to be between 90–425 IU/day, was insufficient in achieving 25OHD >75 nmol/L. Except for one study [73], responses in both 25OHD and 1,25OHD were equally seen [66,70,72], again with the most marked increase in those with the lowest baseline 1,25OHD measurements [66].

Maximal physiologic PTH suppression, however, was inconsistently observed after cholecalciferol supplementation. Whilst four studies demonstrated decreased serum PTH [54,57,67,72], three did not, despite similar cholecalciferol dosing [64,73,77]. Decreased PTH appeared to be in part dependent on participants’ exposure to tenofovir (TDF). Steenhoff *et al*. demonstrated no intergroup difference in PTH between 4,000 and 7,000 IU/day, but without a placebo group for comparison [71]. Although less frequently a reported outcome, changes in serum calcium, phosphate and BAP concentrations were found to be largely unaffected after cholecalciferol supplementation [57,67,73]. Interestingly, in one study, changes in PTH and BAP were only observed in the cholecalciferol supplemented group treated with TDF [67].

Changes in markers of bone remodelling (CTX, P1NP, OC) were not seen in two studies where the treatment arm used doses of 2,000 IU/day or less [67,77] but when the treatment dose was increased to 4,000 IU/day a decrease in CTX and P1NP was observed [77]. These findings are similar to those found in the preliminary data by Sudjaritruk *et al*., however, changes in bone turnover markers were demonstrated in both high- and standard-dose groups without intergroup differences [54]. Increased FGF23 was reported in response to cholecalciferol supplementation, although only in two studies, and increases were only observed in the group receiving TDF-containing ART [57,78].

#### Musculoskeletal outcomes

In the five studies examining bone outcomes, no significant differences in BMD nor BMC were noted between various high- and standard-doses of cholecalciferol versus placebo, despite significant increases in 25OHD. Notably, as demonstrated by Arpadi *et al*., 75% who received supplementation did not consistently maintain serum 25OHD >75 nmol/L; 30% had at least one monthly serum 25OHD concentration <50 nmol/L [56]. Preliminary results of a 48-week randomised open-labelled trial assessing BMD after ‘high-dose’ cholecalciferol (3,200 IU/day) versus ‘normal dose’ (400 IU/day), both groups also supplemented with oral calcium, has recently been presented at the 9th International Workshop on HIV paediatrics [54]. Analyses were stratified by the presence of low lumbar spine BMD Z-score at baseline. BMD gains were seen in both ‘high-dose’ and ‘normal-dose’ groups who had low-BMD at baseline. A weak trend was suggested towards greater BMD gains amongst the ‘high-dose’ group; however, full trial data are awaited. Body size adjustment was not consistently considered, despite recommendations for size adjustment by the ISCD [79], which limits the validity of bone outcomes.

In the one study focused on muscle outcomes, cholecalciferol supplementation did not improve muscle power, force, or strength amongst youth undergoing jumping mechanography, ankle and knee isometric/isokinetic testing and grip strength dynamometry, despite substantial cholecalciferol dosing (7,000 IU/day) which achieved significant increases in 25OHD [62]. However, post-hoc multivariate analysis suggested participants with the greatest increases in 25OHD tended to have greater jump power and energy, although it is unclear if this effect was dependent on baseline 25OHD concentration. Interestingly, in one study, cholecalciferol supplementation did have a beneficial effect on neuromuscular motor skills measured using the Bruininks-Oseretsky Test of Motor Proficiency [80]. This included tests of fine motor precision and integration, dexterity, coordination, balance, and agility. Muscle cross-sectional area was unaffected by cholecalciferol supplementation at the same daily dose in a separate study [57].

Three of four studies examining the effect of cholecalciferol on HAZ provided cholecalciferol as part of a multi-micronutrient supplement and in much lower doses (200–400 IU/day) than the aforementioned studies examining bone and muscle outcomes. No differences in HAZ were observed after 6–18 months on these low-dose regimens [59–61]. Similarly, no differences in HAZ were found in a separate study supplementing 4,000 IU/day to 30 individuals. However, at a much higher dose (7,000 IU/day) this same study found significant differences in HAZ after 12 weeks when compared with 4,000 IU/day [71]. There was again no placebo group for comparison. Furthermore, 32% and 28% of participants, all HIV-positive, in studies by Mda [59] and Steenhoff *et al*. [71] were reported as ‘already stunted’ (HAZ < -2) at baseline. Those supplemented with cholecalciferol in studies by Ndeezi [60] and Chhagan *et al*. [61] also had low mean HAZ at enrolment (HAZ -1.27, -0.9 respectively).

### Risk of bias

**Fig 2:**
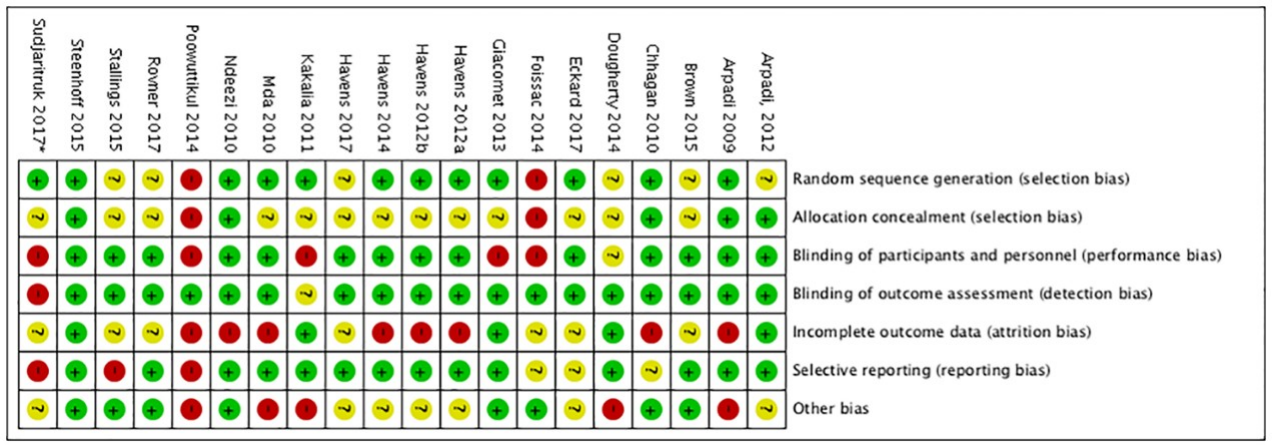
Reviewer assessed risk of bias per Cochrane collaboration domain. High [red], unclear [yellow], and low [green] {Source: created using Review Manager 5.3 [49]}. *Based on avaible data from published abstract, abstract oral presentation, and clinical trials.gov protocol.

The majority of studies were deemed of fair to good quality (Fig 2).

## Discussion

In response to cholecalciferol supplementation, our review found no clear improvements in BMD, BMC, or in muscle power, force or strength. High-dose cholecalciferol appeared to show some effect on HAZ in a single study. The lack of effect on a broad range of musculoskeletal health outcomes may be due to differences in the definition of vitamin D deficiency used as compared to those in place for the general population (S5 Table) and/or the application of these definitions to the variable baseline 25OHD concentrations (Tables 1–4), which allowed enrollment of individuals with 25OHD concentrations which could in some cases already be deemed sufficient, prior to cholecalciferol supplementation.

### The effect of cholecalciferol supplementation on biochemical/endocrine outcomes

The majority of studies aimed to raise 25OHD concentrations >75 nmol/L, intending to supress PTH, although more recent guidelines recommend lower 25OHD concentrations to prevent poor musculoskeletal health in the general population [40,41]. The need for cholecalciferol dosing above that recommended for HIV-negative individuals may be due to reduced intestinal absorption and/or altered vitamin D metabolism, particularly in children and adolescents [31,81]. The omission of those with very low baseline 25OHD biases results against maximal effect; these children and adolescents would largely meet the criteria for rickets/osteomalacia and as such would be expected to benefit most from long-term supplementation. Inclusion of individuals with vitamin D concentrations deemed adequate may have similarly biased results. Alternatively, potentially even higher cholecalciferol doses and thresholds >75 nmol/L for 25OHD may be required. We identified submaximal PTH suppression in 3/7 studies, particularly when lower 25OHD thresholds were used or when 25OHD targets were inconsistently maintained, highlighting the importance of adherence and quality control monitoring in studies. Overall, when assessing for normalisation of biochemical parameters, the studies evaluated seem to favour higher 25OHD targets, *e*.*g*. ≥75 nmol/L.

The Endocrine Society Task Force clinical practice guideline remains one of few to contain recommendations addressing vitamin D deficiency in children and young adults living with HIV, although the evidence-base for these recommendations is limited [42]. Our findings do not dispute their recommendation of a standardised maintenance daily dose of cholecalciferol 2–3 times that recommended for age, even in the absence of universal 25OHD screening. This dose may be simplified to 1,500–2,000 IU/day for all paediatric patients, a recommendation which aligns with optimal dose modelling data by Foissac *et al*. [82]. Higher doses are likely to be required for treatment of documented vitamin D deficiency, and if used, the full safety profile should be substantiated under medical supervision. The Endocrine Society guideline suggests treatment doses of 2,000 IU/day for infants and 4,000 IU/day for children 1–18 years old, which should be accompanied by routine follow-up 25OHD measurements to ensure repletion of biochemical stores. Expert consultation is advocated with higher dosing.

Unfortunately, PTH changes in response to cholecalciferol supplementation were variable. In the five studies addressing bone outcomes, PTH was not measured, not reported, or when reported failed to consistently normalise. Persistently elevated PTH may explain the lack of improvement in BMD/BMC despite increases in 25OHD. Conflicting results in the context of TDF treatment are reported where TDF, by lowering serum 25OHD concentrations, is associated with secondary hyperparathyroidism [83–85]. Havens *et al*. (2012b) observed a reduction in PTH in those receiving TDF and cholecalciferol. Whilst, in those not receiving TDF, PTH values were unchanged despite increased 25OHD, indicating a possible vitamin D independent effect (67]. Dougherty *et al*. showed superior 25OHD responses whilst receiving efavirenz (EFV), but without effects on PTH [72]. This finding contrasts with Eckard *et al*. who demonstrated no differences in 25OHD in those receiving EFV [77]. Likewise, Havens *et al*. (2012a) showed no PTH response from treatment with either TDF or EFV [66]. Potentially, different ART regimens may influence musculoskeletal outcomes, independent of ARTs’ known impacts on vitamin D metabolism [22,23,86].

Low 25OHD concentrations, with secondary hyperparathyroidism, increase bone turnover (*i*.*e*. CTX, P1NP, OC); this is seen in young people with HIV just as it is the wider population [7,87]. Surprisingly, Havens *et al*. (2012b) failed to corroborate this finding, even with normalisation of PTH in the TDF group [67]. Early treatment with cholecalciferol to prevent subclinical hyperparathyroidism may moderate effects on bone turnover. However, this may require high cholecalciferol dosing. Eckard *et al*. only demonstrated changes in CTX and P1NP with 4,000 IU/day (although no PTH response was seen) [77].

FGF23, an important regulator of phosphate homeostasis, is secreted by osteocytes in response to 1,25OHD. FGF23 mediated phosphate regulation in HIV-associated vitamin D deficiency remains poorly understood. TDF is associated with phosphaturia which may perpetuate a hypothesised ‘functional vitamin D deficiency’, explained by higher concentrations of vitamin D binding protein (VDBP) reducing free 1,25OHD [21]. Eckard *et al*. postulated a compensatory decrease in VDBP with EFV after cholecalciferol supplementation [88]. Their findings suggest that TDF and EFV may modify FGF23 response to vitamin D supplementation in adolescents living with HIV by altering vitamin D metabolism, at least in the short term [65]. Further study is needed.

FGF23 increases in renal impairment [89–91], yet only three studies considered renal function in their analysis [67,77,78]. Others excluded patients with renal impairment altogether, or inconsistently measured this at baseline. We further identified heterogeneity in 25OHD assays with five different methods used.

### The effect of cholecalciferol supplementation on musculoskeletal outcomes

The absence of any significant effect of cholecalciferol on BMD/BMC in the five retrieved studies may be a real finding or explained by a number of factors. (i) Intermittent dosing may prove insufficient to sustain steady state 25OHD above essential thresholds. Daily, as opposed to monthly/bi-monthly dosing, may be superior but must be balanced against adherence. However, evidence pertaining to optimal dosing schedules remains contentious, particularly as it relates to effects on bone mass, fractures, and falls [92,93] (ii) Arpadi *et al*.’s exclusion of individuals with the lowest 25OHD measurements may have biased results towards the null, omitting participants who may benefit most from supplementation [56]. (iii) inclusion of individuals with adequate 25OHD concentrations across studies may have equally biased results towards the null, similar to biochemical/endocrine outcomes. (iv) Reported intra-group changes in BMD were not consistently analysed relative to age, changes in body composition (Tanner Staging), and skeletal maturation (particularly height adjustments) [94,95]. Rovner *et al*. [63] was the only study to explicitly state that such height adjustments were performed whereas Arpadi *et al*. was the only study to report supplementary analyses of Tanner stage advancement [56]. (v) DXA measurements are size-dependent, hence size correction is crucial, otherwise low BMD/BMC may be explained by reduced height compared to a control population, rather than an actual deficit on bone mass [79]. BMD must be interpreted relative to stature [96,97].

No paediatric studies from LMIC were available. Promisingly, preliminary results by Sudjaritruk *et al*. represents the first supplementation trial assessing BMD in youth living with HIV outside of the USA; it will be important to see final BMD outcomes correctly size-adjusted and so taking into account growth differences between groups [54]. Furthermore, published data of concurrent changes in biochemical/endocrine markers will be valuable.

Our review highlights a lack of data on muscle size or functional outcomes following cholecalciferol supplementation in HIV-positive youth. Although one study identified increases in neuromuscular motor skills, no effects on muscle power, force or strength were found; perhaps because only 33% achieved 25OH concentrations ≥80 nmol/l. Exploratory *post-hoc* analysis suggested a responder effect such that participants with increased 25OHD after supplementation did show a positive response in jump power and energy [62]. These neuromuscular improvements are important as poor motor function, evaluated by assessing muscular tone, strength, and muscle volume, have been associated with HIV disease progression [98].

The only study to find an effect of cholecalciferol supplementation on HAZ used high-dose supplementation (7,000 IU/day) and examined linear growth as a secondary outcome [71]. HAZ was the primary outcome in just one, much larger study, but of a much younger, heterogenous population with high attrition rates secondary to death [61]. With the exception of the Steenhoff *et al*. trial [71], concurrent 25OHD measurements were not evaluated making it difficult to confirm the extent to which 25OHD concentrations improved after supplementation. In addition, high rates of baseline stunting may represent a missed opportunity, as ‘catch-up’ growth may be unattainable even with adequate micronutrient supplementation. It remains unclear at what age intervention may be beneficial. Follow-up time in all studies was ≤18 months, likely insufficient to detect an effect on HAZ, particularly outside of the peri-pubertal growth period. Lastly, studying cholecalciferol as part of a multi-micronutrient supplement may mean effects are confounded by other micronutrients, supporting the need for placebo controlled studies of high-dose vitamin D supplementation alone.

### Summary of recommendations for future trials of vitamin D supplementation in young people

Moving forward, studies in LMIC are of particular importance. Trials are needed to establish the effect of vitamin D supplementation on musculoskeletal outcomes before PBM is achieved, targeting the key pubertal stages of maximal growth velocity, when impact may be greatest. Supplementation is needed to avoid secondary hyperparathyroidism which is the primary stimulus for bone turnover [7,99]. Hence, concurrent PTH and 25OHD measurements (at appropriate intervals relative to supplementation doses) are needed in studies measuring BMD/BMC and linear growth. Future studies may also wish to investigate practical adjuncts, such as muscle strength training and weight bearing activity. Fracture incidence should be reported in longitudinal studies, at least as a secondary outcome.

Optimal dosing regimens need to be established. Safety profiles need continued evaluation especially at higher doses and for rare drug-related adverse events, missed by smaller studies. Vitamin D should be supplemented at doses and in regimens that aim to provide sustained 25OHD above pre-defined thresholds. Consensus on threshold 25OHD concentrations defining vitamin D deficiency, insufficiency, and sufficiency would be welcome to standardise studies and permit future meta-analyses. The majority of studies employ a 25OHD target of ≥75 nmol/L as sufficient, with values 50–75 nmol/L considered insufficient and <50 nmol/L deficient. We suggest future studies try not to exclude those with the lowest vitamin D concentrations. Whilst, clinical trials where equipoise is lacking are unethical, for example in cases of symptomatic vitamin D deficiency *i*.*e*. rickets, trials in asymptomatic youth who have incidental findings of low 25OHD concentrations which may simply reflect seasonality, can be justified. Where equipoise is lacking, alternative study designs are more appropriate, such as longitudinal study designs evaluating musculoskeletal outcomes before and after cholecalciferol replacement. Studies should also address the long-term effects of supplementation in relation to baseline concentrations, stratifying analysing according to adequacy of baseline 25OHD concentrations.

The combined effect of calcium and vitamin D supplementation on musculoskeletal outcomes in youth living with HIV remains to be established. Future studies need to consistently report and consider the effects of renal function, latitude, season, ethnicity, and local policies on dietary fortification. Lastly, we recommend standardisation of both DXA measurements to take account of size-adjustment as per the revised 2013 ISCD Pediatric Official Position Guideline and the type of 25OHD assay used [79,100]. Use of alternative modalities in the measurement of bone quality such as pQCT, high resolution pQCT and DXA measured trabecular bone score may prove beneficial and should be investigated in this population [101].

## Limitations

Our analysis was limited by the four databases searched and to studies published in English and French. Unfortunately, we were unable to perform a meta-analysis on the available data given the heterogeneity in study designs and populations investigated. This heterogeneity extended to a wide age and geographical range of study particpants, variablity in modes of HIV infection and treatment, and a variety of cholecalciferol supplementation regimes which limits identification of clear patterns in outcomes.

## Conclusion

Our systematic review identified few, small studies, with heterogeneous study designs from which we were unable to draw firm conclusions to guide future evidence-based vitamin D supplementation strategies to optimise musculoskeletal health in youth living with HIV. However, we were able to make a series of recommendations which we feel should be considered by all researchers performing much needed further work in this field. Given the successful role out of universal ART and the transition to HIV-associated chronic disease management, there is an urgent need to identify any interventions that may attenuate the musculoskeletal consequences of a lifetime of HIV infection and treatment.

## Supporting information

S1 Table Complete search strategy (PubMed/MEDLINE). (PDF)

S2 Table Complete search strategy (EMBASE). (PDF)

S3 Table Complete search strategy (CINAHL). (PDF)

S4 Table Complete search strategy (Web of Knowledge). (PDF)

S5 Table Thresholds used to define vitamin D insufficiency, deficiency and excess in the 28 studies reviewed^1, 2^. 1.All values were transformed to nmol/L for standardisation purposes 2.All definitions utilise serum measurements of 25-hydroxyvitamin D_3_. (ND)Not defined.
(PDF)

S1 File PRISMA 2009 checklist. From: Moher D, Liberati A, Tetzlaff J, Altman DG, The PRISMA Group (2009). Preferred Reporting Items for Systematic Reviews and Meta-Analyses: The PRISMA Statement. PLoS Med 6(7): e1000097. doi:10.1371/journal.pmed1000097.
(PDF)
