# Supplementary material for: The impact of vitamin D supplementation on musculoskeletal health outcomes in children, adolescents, and young adults living with HIV: A systematic review
Source: PLoS One. 2018 Nov 15;13(11):e0207022. doi: 10.1371/journal.pone.0207022 (PMC6237309; doi:10.1371/journal.pone.0207022)
Supplement: S1 Table — (PDF) [file pone.0207022.s001.pdf]

S1 Table: Complete search strategy (PubMed/MEDLINE)

| PubMed | Search | Results | Date(DD/MM/YYY)/Time |
|---|---|---|---|
| Subject Query #1<br><br>Children, Adolescence, and Young Adults | (((pediatric* OR paediatric* OR adolescen* OR child* OR "young adult" OR youth* OR minor* OR infant* OR juvenile*))) OR (("pediatrics"[MeSH Terms] OR "adolescent"[MeSH Terms] OR "minors"[MeSH Terms] OR Child OR "young adult"[MeSH Terms] OR "infant"[MeSH Terms])) | 4501271 | 23-12-2017<br><br>18:16 |
| Subject Query #2<br><br>HIV | (((HIV OR "human immunodeficiency syndrome" OR AIDS OR "acquired immunodeficiency syndrome" OR HIV-1 OR HIV-2))) OR (("hiv"[MeSH Terms] OR "acquired immunodeficiency syndrome"[MeSH Terms])) | 428850 | 23-12-2017<br><br>18:17 |
| Subject Query #3<br><br>Vitamin D | ((("vitamin d" OR ergocalciferol* OR "ergocalciferol derivative*" OR calcitriol OR cholecalciferol OR colecalciferol OR calcifediol OR "calcifediol derivative*" OR calcidiol OR "25-hydroxyvitamin d2" OR "25-hydroxyvitamin d" OR "25-hydroxyergocalciferol" OR "1-25 dihydroxycholecalciferol" OR "1-25 dihydroxycholecalciferol" OR "1-25 dihydroxyvitamin d3" OR "calcidiol 1 monooxygenase" OR "vitamin d metabolism")) OR (("vitamin d"[MeSH Terms] OR "ergocalciferols"[MeSH Terms]))) | 77105 | 23-12-2017<br><br>18:18 |
| Combined Query #1 | #1 AND #2 AND #3 | 226 | 23-12-2017<br><br>18:19 |
| Subject Query #4<br><br>Outcome A - Bone Disease | ((("metabolic bone disease*" OR osteoporosis OR "juvenile osteoporosis" OR "primary osteoporosis" OR "childhood-onset primary osteoporosis" OR "idiopathic juvenile osteoporosis" OR osteopenia OR rickets OR osteomalacia OR "vitamin D deficiency" OR "hypovitaminosis D" OR osteolysis OR "bone demineralization" OR "bone demineralisation" OR "pathologic bone demineralization" OR "pathologic bone demineralisation" OR "bone health" OR "musculoskeletal health" OR "skeletal health" OR "skeletal deformity" or "myopathy" or "bone turnover")) OR (("vitamin d deficiency"[MeSH Terms] OR "bone and bones"[MeSH Terms] OR "bone diseases, metabolic"[MeSH Terms] OR "bone diseases, endocrine"[MeSH Terms] OR "bone resorption"[MeSH Terms]))) | 700441 | 12-23-2017<br><br>18:19 |
| Subject Query #5<br><br>Outcome B – Endocrine Markers | ((("parathyroid hormone*" OR PTH OR phosphorus OR phosphate* OR calcium OR "alkaline phosphatase" OR osteocalcin OR PNP-1 OR "procollagen type 1 N-terminal propeptide" OR CTX OR "Collagen type 1 cross-linked C-telopeptide"))) OR (("parathyroid hormone"[MeSH Terms] OR "parathyroid diseases"[MeSH Terms] OR "calcium"[MeSH Terms] OR "calcium-binding proteins"[MeSH Terms] OR "alkaline phosphatase"[MeSH Terms] OR "phosphates"[MeSH Terms])) | 1005411 | 23-12-2017<br><br>18:20 |
| Subject Query #6<br><br>Outcome C – Clinical and Radiographic Findings | ((("Photon Absorptiometry" OR "Dual Energy X-Ray Absorptiometry" OR DEXA OR DXA OR "DEXA scan" OR "bone densit*" OR "bone mineral densit*" OR BMD OR "bone mineral content" OR BMC OR fracture* OR "hand strength" OR "grip strength" OR "muscle strength" OR "bone strength" OR "plate jump" OR "bone demineralisation technique" OR "bone demineralization technique" OR stunt* OR "short stature" OR "growth disorder*" OR "growth restriction" OR "growth delay" OR mechanography OR "bone age" OR "broken bone*")) OR (("absorptiometry, photon"[MeSH Terms] OR "fractures, bone"[MeSH Terms] OR "growth disorders"[MeSH Terms] OR "bone demineralization technique"[MeSH Terms] OR "musculoskeletal physiological phenomena"[MeSH Terms]))) | 1382297 | 23-12-2017<br><br>18:21 |
| Combined Query #2 | #4 OR #5 OR #6 | 2698504 | 23-12-2017<br><br>18:22 |
| Combined Query #3 | (#1 AND #2 AND #3) AND (#4 OR #5 OR #6) | 181 | 23-12-2017<br><br>1823 |
| Limits | Publication Dates(DD/MM/YYYY): (01-01-2000) – (06-07-2016)<br>Language: English, French<br>Species: Human | 150 | 23-12-2017<br><br>18:25 |
