# Supplementary material for: The impact of vitamin D supplementation on musculoskeletal health outcomes in children, adolescents, and young adults living with HIV: A systematic review
Source: PLoS One. 2018 Nov 15;13(11):e0207022. doi: 10.1371/journal.pone.0207022 (PMC6237309; doi:10.1371/journal.pone.0207022)
Supplement: S2 Table — (PDF) [file pone.0207022.s002.pdf]

S2 Table. Complete search strategy (EMBASE)

EMBASE	Search	Results	Date(DD/MM/YYYY)/Time
Subject Query #1<br><br>Children, Adolescence, and Young Adults	((paediatric* or pediatric* or adolescen* or child* or young adult or youth* or minor* or infant* or juvenile*).mp. [mp=title, abstract, heading word, drug trade name, original title, device manufacturer, drug manufacturer, device trade name, keyword] OR (pediatrics/ or juvenile/ or “minor (person)”/ or adolescence/))	4123766	24-12-2017<br><br>20:23
Subject Query #2<br><br>HIV	((HIV or Human Immunodeficiency virus or AIDS or acquired Immunodeficiency Syndrome or HIV-1 or HIV-2).mp. [mp=title, abstract, heading word, drug trade name, original title, device manufacturer, drug manufacturer, device trade name, keyword] OR (acquired immune deficiency syndrome/ OR Human immunodeficiency virus/))	537701	24-12-2017<br><br>20:25
Subject Query #3<br><br>Vitamin D	((vitamin d or ergocalciferol* or ergocalciferol derivative* or calcitriol or cholecalciferol or colecalciferol or calcifediol or calcifediol derivative* or calcidiol or 25-hydroxyvitamin d2 or 25-hydroxyvitamin d or 25-hydroxyergocalciferol or 1-25 dihydroxycholecalciferol or 1-25 dihydroxycholecalciferol or 1-25 dihydroxyvitamin d3 or calcidiol 1 monooxygenase or vitamin d metabolism).mp. [mp=title, abstract, heading word, drug trade name, original title, device manufacturer, drug manufacturer, device trade name, keyword] OR (exp vitamin D/ or exp vitamin supplementation/))	159384	24-12-2017<br><br>20:27
Combined Query #1	#1 AND #2 AND #3	593	24-12-2017<br><br>20:28
Subject Query #4<br><br>Outcome A - Bone Disease	((metabolic bone disease* or osteoporosis or (juvenile adj3 osteoporosis) or (primary adj3 osteoporosis) or (childhood-onset adj3 primary adj3 osteoporosis) or (idiopathic adj3 juvenile adj3 osteoporosis) or osteopenia or rickets or osteomalacia or (vitamin D adj3 deficiency) or hypovitaminosis D or osteolysis or (bone adj3 demineralization) or (pathologic adj3 bone adj3 demineralization) or (bone adj5 health) or (musculoskeletal adj3 health) or (skeletal adj3 health) or (skeletal adj3 deformity) or myopathy or (bone adj3 turnover)).mp. [mp=title, abstract, heading word, drug trade name, original title, device manufacturer, drug manufacturer, device trade name, keyword] OR (exp vitamin D deficiency/ or exp bone disease or bone density/ or bone development/ or bone mineralization/))	1087091	24-12-2017<br><br>20:30
Subject Query #5<br><br>Outcome B – Endocrine Markers	((parathyroid hormone* or PTH or phosphorus or phosphate* or calcium or alkaline phosphatase or osteocalcin or PNP-1 or procollagen type 1 N-terminal propeptide or CTX or collagen type 1 cross-linked C-telopeptide)).mp. [mp=title, abstract, heading word, drug trade name, original title, device manufacturer, drug manufacturer, device trade name, keyword] OR (parathyroid hormone derivative/ or parathyroid disease/ or hyperparathyroidism/ or exp mineral metabolism/ or calcium/ or calcium binding protein/ or alkaline phosphatase/ or phosphate/))	1371176	24-12-2017<br><br>20:34
Combined Query #2	#4 OR #5 OR #6	2623151	24-12-2017<br><br>20:42
Combined Query #3	(#1 AND #2 AND #3) AND (#4 OR #5 OR #6)	294	24-12-2017<br><br>20:43
Limits	Publication Dates: (2000) – (Current)<br>Language: English, French<br>Species: Human	267	24-12-2017<br><br>20:45
Duplicates/Cumulative Total	94	323	
