# Supplementary material for: The impact of vitamin D supplementation on musculoskeletal health outcomes in children, adolescents, and young adults living with HIV: A systematic review
Source: PLoS One. 2018 Nov 15;13(11):e0207022. doi: 10.1371/journal.pone.0207022 (PMC6237309; doi:10.1371/journal.pone.0207022)
Supplement: S3 Table — (PDF) [file pone.0207022.s003.pdf]

S3 Table. Complete search strategy (CINAHL)

CINAHL	Search	Results	Date(DD/MM/YYYY)/Time
Subject Query #1<br><br>Children, Adolescence, and Young Adults	paediatric* or pediatric* or adolescen* or child* or young adult* or youth* or minor* or infant* or juvenile* or (MH "Young Adult") or (MH "Pediatrics") or (MH "Adolescence") or (MH "child") or (MH "Minors (legal)") or (MH "Infant")	655675	24-12-2017<br><br>20:50
Subject Query #2<br><br>HIV	HIV or human immunodeficiency virus or AIDS or acquired immunodeficiency syndrome or HIV-1 or HIV-2 or (MH "Acquired Immunodeficiency Syndrome") or (MH "Human Immunodeficiency Virus")	87818	24-12-2017<br><br>20:50
Subject Query #3<br><br>Vitamin D	vitamin D or ergocalciferol* or ergocalciferol* N3 derivative* or calcitriol or cholecalciferol or calcifediol or calcifediol n3 derivative* or calcidiol or 25-hydroxyvitamin D2 or 25-hydroxyvitamin D or 25-hydroxyergocalciferol or 1-25 dihydroxyvitamin D3 or 1-25 dihydroxycholecalciferol or calcidiol 1 monooxygenase or vitamin D N3 metabolism<br>or (MH "Vitamin D+")	12040	24-12-2017<br><br>20:51
Combined Query #1	#1 AND #2 AND #3	41	24-12-2017<br><br>20:52
Subject Query #4<br><br>Outcome A - Bone Disease	metabolic N3 bone N3 disease* or osteoporosis or juvenile N3 osteoporosis or primary N3 osteoporosis or childhood-onset N3 primary N3 osteoporosis or idiopathic N3 juvenile N3 osteoporosis or osteopenia or rickets or osteomalacia or vitamin D N3 deficiency or hypovitaminosis D or osteolysis or bone N3 demineral i?ation or bone N5 health or musculoskeletal N5 health or skeletal N5 health or myopathy or bone N3 turnover or (MH "Bone Diseases, Metabolic+") or (MH "Bone Resorption+") or (MH "Bone and Bones") or (MH "Vitamin D Deficiency+")	27342	23-12-2017<br><br>20:53
Subject Query #5<br><br>Outcome B – Endocrine Markers	parathyroid hormone* or PTH or phosphorus or phosphate* or calcium or alkaline phosphatase or osteocalcin or PNP-1 or procollagen type 1 N-terminal propeptide or CTX or collagen type 1 cross-linked C-telopeptide or (MH "Parathyroid Hormones") or (MH "Parathyroid Hormones") or (MH "Calcium") or (MH "Calcium Binding Proteins+") or (MH "Alkaline Phosphatase")	27346	24-12-2017<br><br>20:54
Subject Query #6<br><br>Outcome C – Clinical and Radiographic Findings	photon absorptiometry or dual energy X-ray or DEXA or DXA or DEXA N3 scan or bone N3 densit* or bone N3 mineral N3 densit* or BMD or bone N3 mineral N3 content or BMC or fracture* or hand N3 strength or grip N3 strength or muscle N3 strength or bone N3 strength or plate jump or bone N3 demineral i?ation N3 technique or stunt* or short N3 stature or growth N3 disorder* or growth N3 restriction or growth N3 delay or mechanography or bone N3 age or broken N3 bone* or (MH "absorptiometry, Photon") or (MH "Fractures+") or (MH "Musculoskeletal System Physiology+") or (MH "Growth Disorders")	195670	24-12-2017<br><br>20:54
Combined Query #2	#4 OR #5 OR #6	195670	24-12-2017<br><br>20:55
Combined Query #3	(#1 AND #2 AND #3) AND (#4 OR #5 OR #6)	7	23-12-2017<br><br>20:58
Limits	Publication Dates: (01-01-2000) – (31-12-2017)	7	24-12-2017<br><br>20:59
Duplicates/Cumultive Total	1	329	
