# Supplementary material for: The impact of vitamin D supplementation on musculoskeletal health outcomes in children, adolescents, and young adults living with HIV: A systematic review
Source: PLoS One. 2018 Nov 15;13(11):e0207022. doi: 10.1371/journal.pone.0207022 (PMC6237309; doi:10.1371/journal.pone.0207022)
Supplement: S4 Table — (PDF) [file pone.0207022.s004.pdf]

S4 Table. Complete search strategy (Web of Knowledge)

WEB OF SCIENCE	Search	Results	Date(DD/MM/YYYY)/Time
Subject Query #1<br><br>Children, Adolescence, and Young Adults	ts = paediatric* or ts = pediatric* or ts = adolescen* or ts = child* or ts = "young adult" or ts = minor* or ts = infant* or ts = juvenile*	6740579	24-12-2017<br><br>19:54
Subject Query #2<br><br>HIV	ts = HIV or ts = human immunodeficiency virus or ts = AIDS or ts = acquired immunodeficiency syndrome or ts = acquired immune deficiency syndrome or ts = HIV-1 or ts = HIV-2	1795474	24-12-2017<br><br>19:55
Subject Query #3<br><br>Vitamin D	ts = vitamin D or ts = ergolcalciferol* or ts = (ergocalciferol near3 derivative*) or ts = calcitriol or ts = cholecalciferol or ts = colecalciferol or ts = calcifediol or ts = (calcifediol near3 derivative*) or ts = calcidiol or ts = 25-hydroxyvitamin D2 or ts = 25-hydroxyvitamin D or ts = 25-hydroxyergocalciferol or ts = 1-25 dihydroxycholecalciferol or ts = 1-25 dihydroxycholecalciferol or ts = 1-25 dihydroxyvitamin d3 or ts = calcidiol 1 monooxygenase or ts = ("vitamin D" near3 metabolism)	195346	24-12-2017<br><br>19:56
Combined Query #1	#1 AND #2 AND #3	639	24-12-2017<br><br>19:57
Subject Query #4<br><br>Outcome A - Bone Disease	ts = (metabolic near3 bone near3 disease*) or ts = osteoporosis or ts = (juvenile near3 osteoporosis) or ts = (primary near3 osteoporosis) or ts = (childhood-onset near3 primary near3 osteoporosis) or ts = (idiopathic near3 juvenile near3 osteoporosis) or ts = osteopenia or ts = ("vitamin D" near3 deficiency) or ts = hypovitaminosis D or ts = osteolysis or ts = (bone near3 demineralі?ation) or ts = (pathologic near3 bone near3 demineralі?ation) or ts = (bone near5 health) or ts = (musculoskeletal near5 health) or ts = (musculoskeletal near3 health) or ts = (skeletal near3 health) or ts = (skeletal near3 deformity) or ts = myopathy or ts = (bone near3 turnover)	225865	24-12-2017<br><br>19:59
Subject Query #5<br><br>Outcome B – Endocrine Markers	ts = parathyroid hormone* or ts = PTH or ts = phosphorus or ts = phosphate* or ts = calcium or ts = alkaline phosphatase or ts = osteocalcin or ts = PNP-1 or ts = procollagen type 1 N-terminal propeptide or ts = CTX or ts = collagen type 1 cross-linked C-telopeptide	2969359	24-12-2017<br><br>19:59
