# Supplementary material for: The impact of vitamin D supplementation on musculoskeletal health outcomes in children, adolescents, and young adults living with HIV: A systematic review
Source: PLoS One. 2018 Nov 15;13(11):e0207022. doi: 10.1371/journal.pone.0207022 (PMC6237309; doi:10.1371/journal.pone.0207022)
Supplement: S5 Table — 1.All values were transformed to nmol/L for standardisation purposes 2.All definitions utilise serum measurements of 25-hydroxyvitamin D3. (ND)Not defined. (PDF) [file pone.0207022.s005.pdf]

S5 Table. Thresholds used to define vitamin D insufficiency, deficiency and excess in the 28 studies reviewed[1,2]

Study	Vitamin D excess (nmol/L)	Vitamin D sufficiency (nmol/L)	Vitamin D insufficiency (nmol/L)	Vitamin D deficiency (nmol/L)	Severe vitamin D deficiency (nmol/L)
Arpadi *et al.*, 2009 [1]	ND	ND	ND	20-50	<20
Arpadi *et al.*, 2012 [2]	ND	>75	50-75	20-50	<20
Brown *et al.*, 2015 [3]	ND	>79.9	50-79.9	27.5-50	<27.5
Chhagan *et al.*, 2010 [4]	ND	ND	ND	ND	ND
Dougherty *et al.*, 2014 [5]	>400	>79.9	50-79.9	<50	ND
Eckard *et al.*, 2017[6]	>250	>75	<75	ND	ND
Foissac *et al.*, 2014 [7]	>375 (>200 = "unsafe")	>75	25-75	<25	ND
Giacomet *et al.*, 2013 [8]	ND	>75	<75	ND	ND
Havens *et al.*, 2012a,b/2014, 2017 [9-12]	>125	>50	30-50	<30	ND
Kakalia *et al.*, 2011 [13]	ND	>75	25-75	<25	ND
Mda *et al.*, 2010 [14]	ND	ND	ND	ND	ND
Ndeezi *et al.*, 2010 [15]	ND	ND	ND	ND	ND
Poowuttikul *et al.*, 2014 [16]	ND	>87.4	50-87.4	25-50	<25
Rovner *et al.*, 2017[17]	ND	>80	ND	ND	ND
Stallings *et al.*, 2015 [18]	>400	>80	50-79.9	27.5-50	<27.5
Steenhoff *et al.*, 2015 [19]	ND	≥79.9	<79.9	ND	ND

1.All values were transformed to nmol/L for standardisation purposes 2.All definitions utilise serum measurements of 25-hydroxyvitamin D_3. (ND)Not defined

1. Arpadi SM, McMahon D, Abrams EJ, Bamji M, Purswani M, Engelson ES, et al. Effect of bimonthly supplementation with oral cholecalciferol on serum 25-hydroxyvitamin D concentrations in HIV-infected children and adolescents. Pediatrics. 2009;123(1):e121-6.
2. Arpadi SM, McMahon DJ, Abrams EJ, Bamji M, Purswani M, Engelson ES, et al. Effect of supplementation with cholecalciferol and calcium on 2-y bone mass accrual in HIV-infected children and adolescents: a randomized clinical trial. The American journal of clinical nutrition. 2012;95(3):678-85.
3. Brown JC, Schall JI, Rutstein RM, Leonard MB, Zemel BS, Stallings VA. The impact of vitamin D3 supplementation on muscle function among HIV-infected children and young adults: a randomized controlled trial. Journal of musculoskeletal & neuronal interactions. 2015;15(2):145-53.
4. Chhagan MK, Van den Broeck J, Luabeya KK, Mpontshane N, Tomkins A, Bennish ML. Effect on longitudinal growth and anemia of zinc or multiple micronutrients added to vitamin A: a randomized controlled trial in children aged 6-24 months. BMC public health. 2010;10:145.

5. Dougherty KA, Schall JI, Zemel BS, Tuluc F, Hou X, Rutstein RM, et al. Safety and Efficacy of High-Dose Daily Vitamin D3 Supplementation in Children and Young Adults Infected With Human Immunodeficiency Virus. Journal of the Pediatric Infectious Diseases Society. 2014;3(4):294-303.
6. Eckard AR, O'Riordan MA, Rosebush JC, Ruff JH, Chahroudi A, Labbato D, et al. Effects of Vitamin D Supplementation on Bone Mineral Density and Bone Markers in HIV-Infected Youth. Journal of acquired immune deficiency syndromes (1999). 2017;76(5):539-46.
7. Foissac F, Meyzer C, Frange P, Chappuy H, Benaboud S, Bouazza N, et al. Determination of optimal vitamin D3 dosing regimens in HIV-infected paediatric patients using a population pharmacokinetic approach. British journal of clinical pharmacology. 2014;78(5):1113-21.
8. Giacomet V, Vigano A, Manfredini V, Cerini C, Bedogni G, Mora S, et al. Cholecalciferol supplementation in HIV-infected youth with vitamin D insufficiency: effects on vitamin D status and T-cell phenotype: a randomized controlled trial. HIV clinical trials. 2013;14(2):51-60.
9. Havens PL, Hazra R, Stephensen CB, Kiser JJ, Flynn PM, Wilson CM, et al. Vitamin D3 supplementation increases fibroblast growth factor-23 in HIV-infected youths treated with tenofovir disoproxil fumarate. Antiviral therapy. 2014;19(6):613-8.
10. Havens PL, Mulligan K, Hazra R, Flynn P, Rutledge B, Van Loan MD, et al. Serum 25-hydroxyvitamin D response to vitamin D3 supplementation 50,000 IU monthly in youth with HIV-1 infection. The Journal of clinical endocrinology and metabolism. 2012;97(11):4004-13.
11. Havens PL, Stephensen CB, Hazra R, Flynn PM, Wilson CM, Rutledge B, et al. Vitamin D3 decreases parathyroid hormone in HIV-infected youth being treated with tenofovir: a randomized, placebo-controlled trial. Clinical infectious diseases : an official publication of the Infectious Diseases Society of America. 2012;54(7):1013-25.
12. Havens PL, Stephensen CB, Van Loan MD, Schuster GU, Woodhouse LR, Flynn PM, et al. Vitamin D3 Supplementation Increases Spine Bone Mineral Density in Adolescents and Young Adults with HIV Infection Being Treated with Tenofovir Disoproxil Fumarate: A Randomized, Placebo-Controlled Trial. Clinical infectious diseases : an official publication of the Infectious Diseases Society of America. 2017.
13. Kakalia S, Sochett EB, Stephens D, Assor E, Read SE, Bitnun A. Vitamin D supplementation and CD4 count in children infected with human immunodeficiency virus. Journal of Pediatrics. 2011;159(6):951-7.
14. Mda S, van Raaij JMA, MacIntyre UE, de Villiers FPR, Kok FJ. Improved appetite after multi-micronutrient supplementation for six months in HIV-infected South African children. Appetite. 2010;54(1):150-5.
15. Ndeezi G, Tylleskar T, Ndugwa CM, Tumwine JK. Effect of multiple micronutrient supplementation on survival of HIV-infected children in Uganda: a randomized, controlled trial. Journal of the International AIDS Society. 2010;13:18.
16. Poowuttikul P, Thomas R, Hart B, Secord E. Vitamin D insufficiency/deficiency in HIV-infected inner city youth. Journal of the International Association of Providers of AIDS Care. 2014;13(5):438-42.
17. Rovner AJ, Stallings VA, Rutstein R, Schall JI, Leonard MB, Zemel BS. Effect of high-dose cholecalciferol (vitamin D-3) on bone and body composition in children and young adults with HIV infection: a randomized, double-blind, placebo-controlled trial. Osteoporosis International. 2017;28(1):201-9.

18. Stallings VA, Schall JI, Hediger ML, Zemel BS, Tuluc F, Dougherty KA, et al. High-dose Vitamin D-3 Supplementation in Children and Young Adults with HIV A Randomized, Placebo-controlled Trial. Pediatric Infectious Disease Journal. 2015;34(2):E32-E40.
19. Steenhoff AP, Schall JI, Samuel J, Seme B, Marape M, Ratshaa B, et al. Vitamin D(3)supplementation in Batswana children and adults with HIV: a pilot double blind randomized controlled trial. PloS one. 2015;10(2):e0117123.
